# Life-Threatening Hemorrhage, Upper Urinary Tract Extravasation, and Delayed Infection Involving a Persistent Pelvic Collection After Obturator-Route Midurethral Sling Surgery: A Case Report and Narrative Summary of Published Cases

**DOI:** 10.3390/jcm15103875

**Published:** 2026-05-18

**Authors:** In Ae Cho, Yu Jin Lee, Jeesun Lee, Hyen Chul Jo, Jeong Kyu Shin, Won Jun Choi, Jae Yoon Jo

**Affiliations:** 1 Department of Obstetrics and Gynecology, Gyeongsang National University College of Medicine, Jinju 52727, Republic of Korea; dew8274@hanmail.net (I.A.C.); grazysun@gmail.com (J.L.); 73hccho@naver.com (H.C.J.); 2848049@hanmail.net (J.K.S.); choiwj@gnu.ac.kr (W.J.C.); 2 Department of Obstetrics and Gynecology, Gyeongsang National University Hospital, Jinju 52727, Republic of Korea; dbwls9256@naver.com; 3 Institute of Medical Science, Gyeongsang National University, Jinju 52727, Republic of Korea; 4 Department of Obstetrics and Gynecology, Seoul National University College of Medicine, Seoul 03080, Republic of Korea; 5 Department of Obstetrics and Gynecology, Gyeongsang National University Hospital, Changwon 51472, Republic of Korea

**Keywords:** embolization, hemorrhage, stress urinary incontinence, transobturator tape, upper urinary tract extravasation, persistent pelvic collection

## Abstract

**Background/Objectives**: Midurethral sling (MUS) surgery is a standard treatment for stress urinary incontinence in women. Obturator-route MUS procedures reduce retropubic morbidity, but rare concealed hemorrhagic complications can be severe and rapidly progressive. This report describes a complex case of life-threatening hemorrhage, upper urinary tract extravasation, and delayed infection involving a persistent pelvic collection after obturator-route MUS. **Methods**: We reviewed the clinical course, imaging findings, interventions, and follow-up of a 77-year-old woman who developed severe complications after outpatient obturator-route MUS. A descriptive narrative summary of published hemorrhagic complications after TOT or TVT-O procedures was also performed. **Result**: On postoperative day 1, the patient presented with left lower abdominal pain, dizziness, vomiting, tachycardia, and severe anemia. Contrast-enhanced computed tomography showed active bleeding from the left obturator artery, an 11.5 cm pelvic hematoma with bladder displacement, and upper urinary tract contrast extravasation at the left renal pelvis and ureteropelvic junction. Emergency transcatheter arterial embolization and left percutaneous nephrostomy were performed, followed by delayed antegrade double-J ureteral stenting. Four months later, she developed *E. coli* urosepsis with a persistent 7.9 cm paravesical collection. Persistent symptoms despite initial antibiotic therapy required broad-spectrum antibiotics and percutaneous catheter drainage. The drainage fluid was serous, and *S. hominis* isolated from the drainage culture was interpreted as a contaminant; therefore, the collection was managed as a clinically suspected infection involving a persistent pelvic collection rather than as a microbiologically confirmed infected hematoma. **Conclusions**: After obturator-route MUS, severe abdominal or pelvic pain, dizziness, tachycardia, hypotension, or abrupt hemoglobin decline should prompt contrast-enhanced CT to evaluate for concealed pelvic arterial bleeding and associated urinary tract extravasation. Early multidisciplinary coordination and follow-up of persistent pelvic collections may be important in complex cases.

## 1. Introduction

Stress urinary incontinence (SUI) is prevalent among women, with its burden increasing with age and obesity [1]. Midurethral sling (MUS) surgery is a standard treatment because it is minimally invasive, often performed as a day-case procedure under local or regional anesthesia, with high continence rates. Retropubic tension-free vaginal tape (TVT) was introduced in the mid-1990s and has since been widely adopted. Transobturator techniques were subsequently developed to avoid blind needle passage through the retropubic space. Delorme described the outside-in transobturator tape (TOT) in 2001, and de Leval introduced the inside-out TVT-Obturator (TVT-O) approach in 2003 [2,3]. Although both TOT and TVT-O use the obturator route, their trocar trajectories are not identical. A cadaveric study comparing outside-in and inside-out transobturator tape procedures showed that inside-out tapes passed significantly closer to the obturator canal than outside-in tapes, although whether this anatomical difference translates into a clinically meaningful difference in vascular injury risk remains uncertain [4]. Therefore, in the present report, we use the broader term “obturator-route MUS” when the exact trajectory cannot be verified. Since then, transobturator slings have been increasingly used in routine practice, and retropubic and transobturator approaches have been compared in many reviews [5,6,7].

More recent systematic evidence, including the updated Cochrane review by Ford et al., supports broadly similar short- and medium-term effectiveness between retropubic and transobturator MUS approaches, while showing different adverse-event profiles [7]. Retropubic slings are more commonly associated with bladder perforation and voiding dysfunction, whereas transobturator slings are associated with groin pain and obturator-route complications; severe vascular injury remains rare and is mainly described in case reports [5,7]. Accordingly, when discussing obturator-route complications, it is important not to assume that TOT and TVT-O are anatomically interchangeable in all respects. Most bleeding events after TOT or TVT-O are self-limiting and can be conservatively managed in hemodynamically stable patients [8]. However, findings from case reports show uncommon severe hemorrhage with hypotension, tachycardia, and marked hemoglobin decline due to pelvic or vulvar hematoma, sometimes requiring transfusion and urgent hemostasis, including packing, surgical exploration, or selective pelvic arterial embolization [9,10]. To contextualize the present report, we reviewed published case reports of hemorrhagic complications after obturator-route MUS (TOT and TVT-O), summarizing the most frequently implicated vascular structures and the spectrum of clinical presentations. Herein, we describe a severe and complex complication following an outpatient obturator-route MUS procedure: acute massive hemorrhage with concomitant upper urinary tract extravasation, followed by delayed infection involving a persistent paravesical collection approximately 4 months later.

## 2. Case Presentation

A 77-year-old Korean woman (gravida 6, para 6; all vaginal deliveries) was transferred to the emergency department (ED). Her medical history included a total vaginal hysterectomy performed approximately 15 years earlier for uterine prolapse. She had been diagnosed with cardiac arrhythmia and was receiving an angiotensin receptor–neprilysin inhibitor.

One day before transfer, she underwent an outpatient obturator-route midurethral sling procedure, reported as a TOT procedure, under local anesthesia at a private clinic for SUI. She was discharged after confirmation of spontaneous voiding. The original operative report was unavailable; therefore, the sling manufacturer, mesh material, exact trocar trajectory, and distinction between outside-in TOT and inside-out TVT-O could not be confirmed. Information regarding the surgeon’s case volume or experience was also unavailable.

On postoperative day 1, she developed left lower abdominal pain accompanied by dizziness and vomiting and presented to a nearby ED. Her heart rate was 159 beats/min, and laboratory tests revealed severe anemia (hemoglobin, 6.2 g/dL; hematocrit, 19.2%), with preserved renal function (blood urea nitrogen [BUN]/creatinine, 22/0.78 mg/dL). She received two units of packed red blood cells (PRBCs). Because interventional management was not available at that facility, she was transferred to our ED.

**Figure 1:**
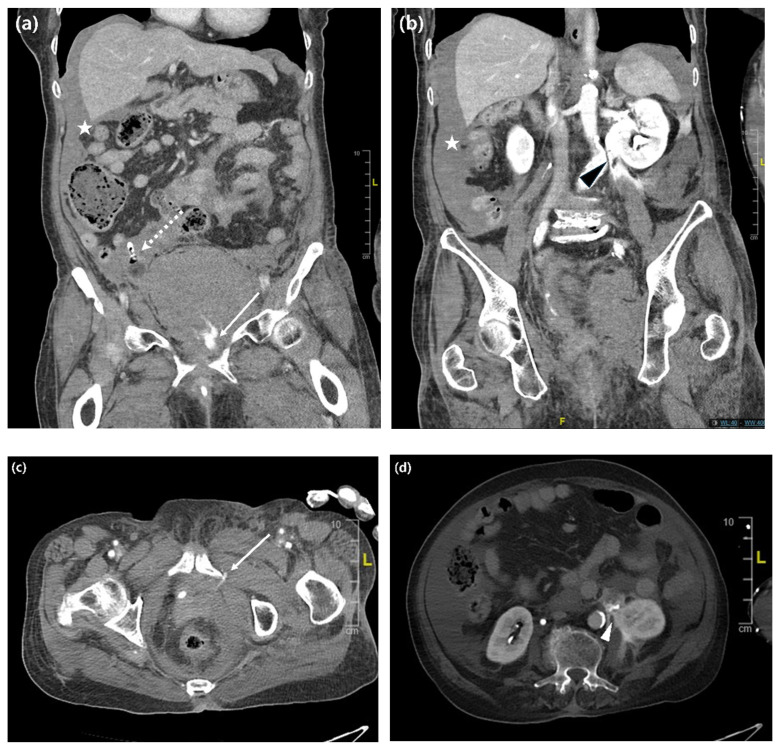
Initial enhanced computed tomography findings after the transobturator tape procedure. Coronal view of delayed phase, (**a**); coronal view of portal phase, (**b**); axial view of portal phase, (**c**); axial view of portal phase, contrast enhanced, (**d**). White asterisk: hemoperitoneum; white dotted arrow: Foley catheter; white solid arrow: active contrast extravasation from the left obturator artery; black arrowhead and white arrowhead: contrast extravasation at the left renal pelvis/ureteropelvic junction (UPJ) level.

On arrival, her vital signs were as follows: blood pressure, 80/58 mmHg; heart rate, 80 beats/min; respiratory rate, 18 breaths/min; temperature, 36.8 °C; and oxygen saturation, 99% on room air. Her hemoglobin level was 9.8 g/dL, and hematocrit was 29.0%. Early resuscitation markers included serum lactate 3.40 mmol/L, arterial blood gas values of pH 7.48, PaCO_2_ 26 mmHg, and bicarbonate 19 mmol/L, with an estimated base deficit of approximately 3.9 mmol/L, mild azotemia (BUN/creatinine, 22.1/1.14 mg/dL), and limited early urine output after arrival with an indwelling Foley catheter already in place (70 mL at 20:00, 25 mL at 21:00, and 50 mL at 22:00; 19:00 output not documented). Contrast-enhanced computed tomography (CT) revealed active arterial bleeding from the left obturator artery, a large pelvic hematoma measuring 11.5 cm with displacement of the urinary bladder, and contrast extravasation at the level of the left renal pelvis and ureteropelvic junction (UPJ) (Figure 1a–d). While receiving an additional three units of PRBCs, she underwent emergency transcatheter arterial embolization and left percutaneous nephrostomy (PCN) for suspected upper urinary tract extravasation at the UPJ level. Thus, a total of five units of PRBCs were transfused during the acute hemorrhagic phase: two units before transfer at the referring hospital and three additional units during emergency management at our institution. She was admitted to the Department of Gynecology for intravenous antibiotic administration and close observation.

After embolization, her abdominal pain and vomiting improved. On the following day, hemoglobin levels and hematocrit were 11.1 g/dL and 32.0%, respectively, and mild azotemia was noted (BUN/creatinine, 24.5/0.96 mg/dL). A urology consultation was requested to assess the feasibility of retrograde double-J ureteral stenting. Cystoscopy was not attempted because, based on CT findings, the urology team judged that pelvic hematoma-related bladder deviation would likely result in poor ureteral-orifice visualization and technically difficult retrograde access. Therefore, the previously placed left PCN was maintained for urinary diversion and delayed antegrade double-J ureteral stent placement through the nephrostomy tract was planned. Seven days after the initial hemorrhagic event, an antegrade double-J ureteral stent was successfully inserted through the nephrostomy tract. Follow-up contrast-enhanced CT showed no further contrast extravasation and a reduction in the pelvic hematoma size; therefore, the PCN was removed, whereas the double-J ureteral stent was left in place. After PCN removal, laboratory results showed a white blood cell count (WBC) of 10,680/µL, hemoglobin level of 12.8 g/dL, hematocrit of 40.0%, and BUN/creatinine levels of 9.7/0.61 mg/dL. The patient was discharged following clinical improvement. No postoperative neurological deficit was documented, including groin pain, thigh sensory disturbance, motor weakness, gait disturbance, or symptoms suggestive of obturator or pudendal nerve injury. Approximately 4 months later, she presented to a local ED with fever and lower abdominal pain. The exact duration of fever and lower abdominal pain before presentation to the local ED was not documented in the available outside-hospital records. The double-J ureteral stent had been removed 8 weeks before the delayed infectious episode. CT revealed a 7.9 cm paravesical cystic mass. Laboratory tests showed leukocytosis (WBC, 14,200/µL), markedly elevated C-reactive protein (CRP, 170 mg/L) and procalcitonin (8.69 µg/L), and mild renal impairment (BUN/creatinine, 33.6/1.11 mg/dL). *Escherichia coli* was isolated from blood and urine cultures, and she was treated for urosepsis with intravenous ceftriaxone for 5 days. Because lower abdominal pain and a paravesical cystic lesion persisted, she was transferred to our hospital for further management.

**Figure 2:**
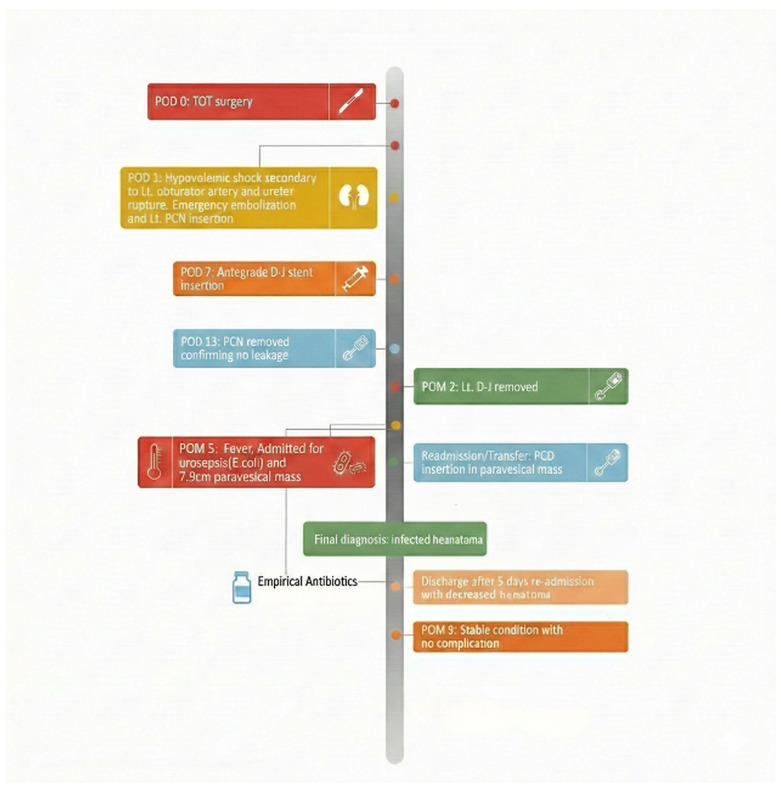
Clinical course and management timeline of the patient. POD, postoperative day; POM, postoperative month; PCN, percutaneous nephrostomy; D-J, double-J ureteral stent; PCD, percutaneous catheter drainage; TOT, transobturator tape.

At our institution, the pelvic cystic lesion was considered most consistent with an abscess or urinoma. Despite being afebrile, the patient experienced persistent lower abdominal discomfort, with no interval change in the cystic mass. Before drainage-culture results were available, antibiotic therapy was empirically escalated to intravenous piperacillin–tazobactam because of persistent symptoms and a residual pelvic collection despite 5 days of ceftriaxone, prior antibiotic exposure, recent hospitalization, and concern for healthcare-associated complicated urinary tract infection or secondary infection involving the persistent collection. Percutaneous catheter drainage (PCD) was then performed by interventional radiology. The drained fluid was serous. Culture of the PCD aspirate yielded *Staphylococcus hominis*. Infectious disease consultation considered *S*. *hominis* unlikely to be the causative pathogen because it is a common skin commensal and the drained fluid was serous. Instead, the previously documented *E*. *coli* bacteremia and bacteriuria were considered more consistent with the patient’s urosepsis and persistent inflammatory course. Although secondary infection of the residual hematoma was not microbiologically confirmed, the collection was treated as a clinically suspected infected hematoma because of the persistent collection, elevated inflammatory markers, clinical course, and improvement after drainage with antibiotic-supported management. After approximately 5 days of drainage and antibiotic therapy, follow-up evaluation confirmed a reduction in the collection size, with improvement in inflammatory markers and renal function (WBC 6800/µL, CRP 1.1 mg/L, BUN/creatinine 14/0.78 mg/dL). The patient was discharged in a stable condition. She remained free of further complications during the follow-up at 21 months after the second discharge. The patient’s clinical course and management timeline are summarized in Figure 2.

### 2.1. Descriptive Narrative Summary of Published Cases

To contextualize the present case, we performed a descriptive narrative summary of published reports and small case series describing hemorrhagic complications after obturator-route midurethral sling procedures. This summary was intended to illustrate the clinical spectrum of reported cases rather than to provide a systematic review, incidence estimate, laterality analysis, or comparative risk assessment. Therefore, PRISMA-NR or other formal systematic review reporting standards were not applied. PubMed and Google Scholar were searched from January 2001, when the transobturator approach was first introduced, to December 2025. Search terms included “transobturator tape,” “TOT,” “TVT-O,” “midurethral sling,” “obturator sling,” “hemorrhage,” “hematoma,” “vascular injury,” “arterial bleeding,” “embolization,” “obturator artery,” and “internal pudendal artery.” Reference lists of relevant articles were also screened manually.

We included English-language case reports or small case series describing clinically significant hemorrhage, hematoma, or vascular injury after outside-in TOT or inside-out TVT-O procedures when sufficient clinical information was available to characterize presentation, imaging findings, management, or outcome. Reports involving retropubic TVT alone, TVT-Secur, other single-incision mini-slings, non-obturator sling routes, non-hemorrhagic mesh complications, or unclear sling trajectories were excluded. Extracted variables were summarized descriptively and included patient age, procedure type, time to presentation, symptoms, bleeding source, hematoma location and size, imaging modality, transfusion requirement, time to intervention, management category, and outcome. When a variable was not explicitly reported in the source article, it was coded as NR rather than inferred. No quantitative pooling, formal risk-of-bias assessment, or comparative risk/effectiveness assessment was attempted. Because case ascertainment was based on published reports and manual reference screening, incomplete retrieval and publication bias cannot be excluded.

### 2.2. Discussion and Literature Review

Obturator-route MUS procedures, including outside-in TOT and inside-out TVT-O, were developed to reduce retropubic-space morbidity while maintaining continence efficacy. Although these procedures are generally safe, severe hemorrhagic complications can still occur because the trocar pathway passes through a confined anatomic region containing obturator, internal pudendal, and internal iliac vascular branches. The present case is clinically notable because of a life-threatening obturator arterial hemorrhage, concomitant upper urinary tract extravasation, and delayed infection involving a persistent paravesical collection that occurred in temporal sequence after an outpatient obturator-route MUS procedure. Table 1 summarizes reported hemorrhagic complications after TOT or TVT-O.

The many NR entries in Table 1 reflect the inconsistent reporting quality of the available case reports. Key variables such as exact bleeding source, hemodynamic status, transfusion requirement, and time to intervention were not uniformly reported. Therefore, Table 1 should be interpreted only as a descriptive map of published presentations and management strategies, not as evidence for incidence, laterality, procedural risk, or comparative effectiveness. Despite these limitations, the reported cases suggest a clinically useful distinction: stable localized hematomas were generally managed conservatively, whereas hemodynamic instability, abrupt hemoglobin decline, enlarging hematoma, or active arterial extravasation prompted embolization, surgical exploration, or drainage. Thus, management appears to be driven more by physiologic instability and imaging evidence of active bleeding than by the reported sling subtype or bleeding side.

The main novelty of our case is not simply the occurrence of hemorrhage after an obturator-route sling, which has been reported previously, but a temporal coexistence of life-threatening pelvic arterial bleeding, upper urinary tract extravasation, and a delayed infection involving a persistent paravesical collection. Severe obturator arterial bleeding can occur even after an outpatient procedure under local anesthesia and may present with hypotension, tachycardia, profound anemia, and minimal external bleeding. Therefore, disproportionate abdominal or pelvic pain, dizziness, tachycardia, hypotension, or an abrupt hemoglobin decrease after obturator-route MUS should prompt urgent evaluation for concealed pelvic bleeding.

The mechanism of upper urinary tract extravasation in this patient remains unconfirmed. Several explanations may be considered, including direct ureteral injury, transient distal ureteral obstruction caused by pelvic hematoma or bladder–trigone displacement, functional obstruction without persistent anatomic narrowing, or other unrecognized perioperative factors. Direct ureteral injury by an obturator-route trocar was considered anatomically less likely because no definite contrast extravasation was identified from the distal ureter or bladder on available CT images. However, it could not be definitively excluded because the original operative report was unavailable, the exact trocar trajectory could not be confirmed, and ureteral integrity was not directly visualized at the time of the acute event. Hematoma-related distal ureteral compression remains a biologically plausible explanation for the upper urinary tract extravasation observed in this patient. Analogous case reports have described ureteral obstruction, renal forniceal rupture, or urogenic sepsis associated with compressive pelvic or retroperitoneal hematomas [20,21]. However, this remains a hypothesis supported by analogous cases and unconfirmed in this specific patient. In particular, delayed-phase CT urography documenting the degree and level of ureteral obstruction was not available. Therefore, the imaging findings should be interpreted as upper urinary tract extravasation temporally associated with a large pelvic hematoma, rather than definitive proof that the hematoma caused distal ureteral compression and UPJ rupture.

The delayed infectious episode should also be interpreted cautiously. The patient had *E*. *coli* bacteremia and bacteriuria, persistent inflammatory markers, and a residual paravesical collection that improved after drainage and antibiotic-supported management. However, secondary infection of the residual hematoma was not microbiologically confirmed, and *S. hominis* isolated from the drainage culture was interpreted as a contaminant. Therefore, this episode is more appropriately described as *E*. *coli* urosepsis associated with a persistent pelvic collection managed as possible secondary involvement of the collection, rather than as a proven infected hematoma. Piperacillin–tazobactam was selected empirically before the drainage-culture result was available because of persistent symptoms after ceftriaxone, prior antibiotic exposure, recent hospitalization, and concern for healthcare-associated complicated urinary tract infection or secondary infection involving the persistent collection. Several practical clinical messages follow from this case. First, contrast-enhanced CT should be considered promptly when severe pain, dizziness, tachycardia, hypotension, or abrupt hemoglobin decline occurs after obturator-route MUS, even in the absence of obvious vaginal bleeding. Second, CT should be evaluated not only for active arterial extravasation and hematoma size, but also for bladder displacement, hydronephrosis, ureteral compression, and urinary contrast extravasation. Third, when vascular bleeding and urinary tract extravasation coexist, early multidisciplinary coordination among gynecology, interventional radiology, and urology is essential for hemostasis, urinary diversion, and follow-up imaging. Finally, a persistent pelvic collection after major hemorrhage warrants follow-up because delayed infection or secondary involvement during a later urinary infection may occur even after initial stabilization.

This report has several limitations. Because the original operative report was unavailable, the sling manufacturer, mesh material, exact trocar trajectory, and distinction between outside-in TOT and inside-out TVT-O could not be confirmed. Most importantly, the proposed mechanism of hematoma-related distal ureteral compression remains unconfirmed. Delayed-phase CT urography documenting the degree and level of obstruction was not available, and ureteral integrity was not directly visualized at the time of the acute event. Therefore, the observed upper urinary tract extravasation cannot be attributed definitively to hematoma-related distal ureteral compression. Direct ureteral injury, transient functional obstruction, trigonal distortion, or other unrecognized factors cannot be completely excluded. The delayed infectious episode was clinically suspected but was not microbiologically proven to involve residual hematoma. The utility of Table 1 is limited by the heterogeneous and incomplete reporting of the original case reports; therefore, variables not explicitly described were coded as NR and should not be interpreted as absent. Accordingly, apparent patterns in bleeding source, timing, management, or outcome should be interpreted cautiously and considered descriptive and hypothesis-generating rather than confirmatory.

In conclusion, concealed hemorrhage after obturator-route MUS can be severe and may coexist with urinary tract extravasation or delayed complications involving a persistent pelvic collection. The proposed hematoma-related obstructive mechanism is biologically plausible but remains unconfirmed in this patient, and alternative explanations cannot be excluded. This case should be regarded as hypothesis-generating and clinically instructive, emphasizing careful reassessment when postoperative deterioration occurs after obturator-route MUS.

## Figures and Tables

**Table 1:** Summary of reported hemorrhagic complications after TOT or TVT-O procedures.

Case	Ref.	Age	Procedure	Time to Presentation	Symptoms/Signs	HI	Bleeding Source	Hematoma Size/Imaging	Transfusion	Time to Intervention	Management Category	Outcome
1	[11]	57	Transobturator sling	POD 6	Left hip pain, diffuse bruising in left hip, umbilicus and inner thigh	No	NR	4.2 × 5.7 cm/CT	NR	NA; conservative	Conservative	Hematoma decrease
2	[11]	47	Transobturator sling	POD 7	Vaginal bleeding	No	NR	7.4 × 7.9 cm/CT	NR	After recurrent bleeding despite packing; exact interval NR	Surgical (CT-guided drainage)	Hematoma decrease
3	[12]	late 40s	Transobturator suburethral tape	POD 5	Abdominal pain	No	NR	8.1 × 7.0 × 5.1 cm/CT	NR	NA; conservative	Conservative	Hematoma decrease
4	[13]	38	TVT-O	5 h	Vulvar pain; swelling	No	NR	NR/clinical diagnosis	No	NA; conservative	Conservative	Hematoma decrease
5	[14]	61	TVT-O	3 h	Right upper thigh pain	Yes	Arterial bleeding in cavum Retzii mediolateral vessel to obturator nerve	10cm/US	Yes, 2 PRBC units	Same day; exact interval NR	Surgical (laparotomy)	Hematoma decrease
6	[15]	56	Transobturator sling	POD 3	Suprapubic discomfort; nausea	No	Retzii space; specific vessel NR	8 × 10 × 11 cm/CT	No	NA; conservative	Conservative	Hematoma decrease
7	[10]	42	Outside-in transobturator sling	<1 h	Vaginal bleeding	No	Suspected venous or accessory obturator vessel injury	NR/CT	NR	Intraoperative; after failed standard packing	Surgical (QuikClot packing)	Clinically improved
8	[16]	46	TVT-O	2 h	Left vulvar pain	No	Anterior division of left obturator artery	NR/angiography	Yes, 2 PRBC units	Same day; exact interval NR	Endovascular (embolization)	Clinically improved
9	[9]	33	TOT, outside-in	1 day	Perineal pain and vaginal bleeding	Yes	Left internal pudendal artery	7 cm/angiography	Yes, 4 PRBC units	POD 1; exact interval NR	Endovascular (embolization)	Hematoma decrease
10	[17] ^a^	44	TOT	POD 1	Abdominal pain, voiding difficulty, vaginal bleeding	No	Upper vaginal branch of left internal iliac artery	NR/angiography	Yes, 2 PRBC units	POD 1; exact interval NR	Endovascular (embolization)	Hematoma decrease
11	[17] ^a^	43	TOT	7 h	Abdominal pain and tenderness	Yes	Pubic branch of left obturator artery; left inferior epigastric artery	10 cm/CT + angiography	Yes, 8 PRBC units	Same day after presentation; exact interval NR	Endovascular (embolization and laparotomy)	Hematoma decrease
12	[18]	54	TOT	4 h	Abdominal pain and distension	Yes	Branch of left obturator artery	12.4 × 7.9 × 8.9 cm/CT + angiography	Yes, units NR	Same day; exact interval NR	Endovascular (embolization)	Hematoma regression
13	[8]	49	TOT, as reported by source	POD 1	Left thigh pain and swelling	No	NR	4 × 4 cm/US + MRI	No	NA; conservative	Conservative	Hematoma regression
14	[19]	35	TOT, outside-in	12 h	Right thigh erythema and pain; fever	No	NR	NR/MRI	NR	After failed antibiotics; exact interval NR	Surgical (abscess drainage)	Symptoms resolved

TOT, transobturator tape; TVT-O, tension-free vaginal tape-obturator; POD, postoperative day; HI, hemodynamic instability; CT, computed tomography; MRI, magnetic resonance imaging; US, ultrasonography; PRBC, packed red blood cells; NR, not reported; NA, not applicable. HI was recorded as “Yes” when hypotension, shock, syncope, marked tachycardia requiring resuscitation, or explicit hemodynamic instability was reported. Time to intervention refers to the interval from symptom recognition or presentation to definitive hemostatic or drainage intervention. Management category was classified as conservative, surgical, or endovascular according to the primary definitive treatment modality. Unreported variables after full-text review were recorded as NR and should not be interpreted as absent. Only obturator-route MUS cases, including outside-in TOT and inside-out TVT-O, were included; retropubic TVT, TVT-Secur, other single-incision mini-slings, non-obturator routes, and unclear sling trajectories were excluded. ^a^ From this report, only cases involving transobturator-route sling procedures were included; cases involving single-incision mini-slings, such as TVT-Secur, or cases with an unclear sling trajectory were excluded.

## Data Availability

Data sharing is not applicable to this article as no datasets were generated or analyzed during the current study.
